# The association between miR-423 rs6505162 polymorphism and cancer susceptibility: a systematic review and meta-analysis

**DOI:** 10.18632/oncotarget.16319

**Published:** 2017-03-17

**Authors:** Ru Chen, Yonglan Zheng, Lin Zhuo, Shengfeng Wang

**Affiliations:** ^1^ National Cancer Center/Cancer Hospital, Chinese Academy of Medical Sciences and Peking Union Medical College, Beijing, China; ^2^ Department of Medicine, University of Chicago, Chicago, IL, USA; ^3^ Department of Epidemiology and Bio-statistics, School of Public Health, Peking University Health Science Center, Beijing, China

**Keywords:** miR-423, rs6505162, polymorphism, cancer susceptibility, meta-analysis

## Abstract

The association between miR-423 polymorphism (C > A) and the risk of different cancers are still controversial. We performed a meta-analysis to clarify its association with multiple cancer risks. PubMed and Embase (as of 10th September, 2016) were searched. A total of 17 studies from 16 articles, consisting of 8,582 cases and 10,291 controls, were finally qualified and enrolled in this meta-analysis. The pooled results showed that the miR-423 AA genotype was associated with decreased cancer risk under the recessive model (odds ratio [OR] = 0.87, 95% confidence interval [CI]: 0.78~0.98, *P* = 0.020). However, this association became non-significant after excluding the study with the smallest odds ratio. Subgroup analyses revealed a significant decrease in risk of lung cancer (dominant model: OR = 0.73, 95 % CI: 0.60~0.89, *P* = 0.002; recessive model: OR = 0.59, 95 % CI: 0.37~0.95, *P* = 0.031). Our study indicates that miR-423 rs6505162 might be associated with a reduced risk of cancers, however, this finding need to be evaluated further in larger samples, especially subgroup analyses. In addition, cancer-specific functional studies are especially needed to reveal the underlying mechanisms between miR-423 and the etiology of cancer.

## INTRODUCTION

Cancer poses a major threat to public health worldwide, and its burden continues to increase [1]. The causal association between genetic alterations and cancer is supported by extensive experimental and epidemiological data [2]. MicroRNAs (miRNAs) are a class of endogenous, non-coding, single-stranded RNAs approximately 22 nucleotides in length. microRNAs guide RNA-induced silencing complex to the miRNA recognition elements of the targeted protein-coding transcripts or other competitive endogenous RNAs, and thereby play a role in post-transcriptional regulation [3–6]. The target genes cover about one-third of the human genome, including genes involved in cell division, growth, differentiation, proliferation and apoptosis [7]. Over 50% of miRNAs genes are located in cancer-associated genomic regions or fragile sites, and have been found to be involved in carcinogenesis as tumor suppressors or oncogenes [8, 9]. Evidence also exists to support the abnormal expression of various miRNAs in the cancer development [7, 10]. Therefore, it is reasonable that single-nucleotide polymorphisms (SNPs) in miRNAs genes might alter miRNAs expression and maturation. These SNPs can alter the effects of miRNAs on their target genes, possibly leading to abnormal biological metabolism and modified cancer susceptibility [8, 11, 12].

The rs6505162 SNP is located in the *pre-miR-423* and maps to 17q11.2, with a nucleotide alteration from C to A. The aberrant expression of both mature forms of the miR-423 (named as miR-423-3p and miR-423-5p) has been observed in numerous cancer types [13–17]. A growing number of studies also have been conducted to assess this polymorphism's association with the risk of different cancers, but the results are conflicting rather than conclusive [18–33]. About ten of 17 published studies have shown no correlation between rs6505162 and risk of different cancers [18–21, 23, 25, 26, 29, 31, 33]. For the remaining studies with significant results, the same allele of A was found to be risky in 3 studies, [22, 24, 28] but to be protective in 4 studies [26, 27, 30, 32]. Given the requirement of risk classification in populations, [34] we performed this systematic review and meta-analysis to improve evaluation of the association between miR-423 rs6505162 polymorphism and multiple cancer risks.

## RESULTS

### Study selection

**Figure 1:**
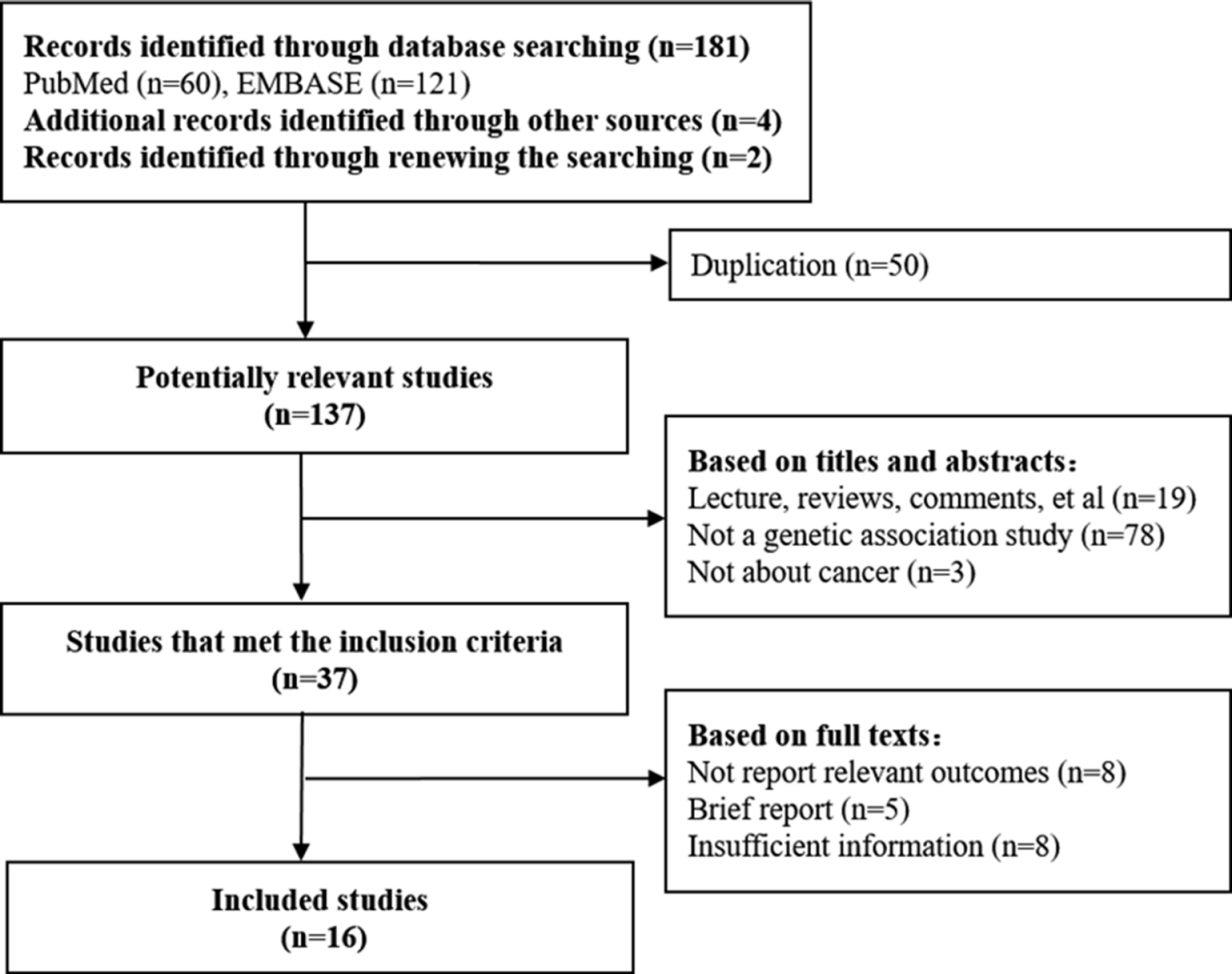
Flow diagram of the results of the search strategy

The selection flow of studies was summarized in Figure 1. The initial search identified 187 articles on cancer risk and/or clinical outcome assessment for miR-423 rs6505162. According to the inclusion criteria, 16 articles were included. One of the articles reported two ethic groups separately and thus was divided into two studies.

### The characteristics of included studies

All of the eligible studies were issued in English except for one issued in Chinese [33]. A total of 8,582 cases and 10,291 controls were involved in the pooled analyses. The publication year of selected studies ranged from 2008 to 2016. The ethnicities studied included Caucasian, Asian, Black and mixed populations. Table 1 presents the primary characteristics and quality assessment of the included studies. The A allele of rs6505162 was indicated as risk factor in 3 studies, but protective factors in 4 studies. All the remaining was not significant. The samples used for the examination of rs6505162 were extracted from blood in all included studies. Hardy-Weinberg equilibrium (HWE) tests were performed in all but one study, [29] and the genotyping distribution was in agreement with HWE in these studies. The quality of primary studies assessed by Newcastle-Ottawa Scale (NOS) showed that all studies were rated as “high quality” except for one [22].

**Table 1:** Baseline characteristics of eligible studies

Author	Year	Country	Ethnicity	Diagnosis	Sample Size		Genotyping	NOS Score	Effect in original study (for A allele of rs6505162)
					Case	Control			
Yang^32 α^	2008	USA	Caucasians	Bladder cancer	746	746	SNPlex	8	Protective
Ye^27^	2008	USA	Caucasian	Esophageal cancer	346	346	Taqman	8	Protective
Smith^24^	2012	Australia	Caucasian	Breast cancer	179	174	HRM	7	Risk
Umar^25^	2013	India	Indian	Esophageal cancer	289	309	PCR-RFLP	8	Ns
Wang^26 α, β^	2013	SouthAfrica	Black	Esophageal squamous cell carcinoma	368	583	SNPlex	7	Protective
			Mixed		197	420		8	Ns
Ma^20^	2013	China	Chinese	Breast cancer (triple negative)	191	192	MassARRAY	8	Ns
Yin^28 β^	2013	China	Chinese	Esophageal squamous cell carcinoma	629	686	Taqman	9	Risk
Ma^21 α, β^	2014	China	Chinese	Hepatocellular carcinoma	984	991	MassARRAY	7	Ns
Yin^29 β^	2015	China	Chinese	Lung cancer	258	310	Taqman	8	Ns
Zhang^33^	2015	China	Chinese	breast cancer	384	192	MassARRAY	7	Ns
Zhu^31 α, β^	2015	China	Kazakh	Esophageal squamous cell carcinoma	248	300	MassARRAY	8	Ns
He^18 α^	2015	China	Chinese	Breast cancer	450	450	MassARRAY	8	Ns
Yin^30 α^	2016	China	Chinese	Lung cancer	575	608	SNPlex	8	Protective
Jiang^19 α, β^	2016	China	Chinese	Gastric cancer	898	992	MassARRAY	9	Ns
Shen^23 α, β^	2016	China	Chinese	Esophageal squamous cell carcinoma	1400	2185	SNaPshot	8	Ns
Morales^22^	2016	Chile	Chilean	Breast cancer	440	807	Taqman	5	Risk

Note: Ns, not significant; NOS Score, Newcastle-Ottawa Scale score.

^α^we could extract the related data to calculate the adjusted odds ratio under dominant model;

^β^we could extract the related data to calculate the adjusted odds ratio under recessive model.

### Meta-analysis

**Figure 2:**
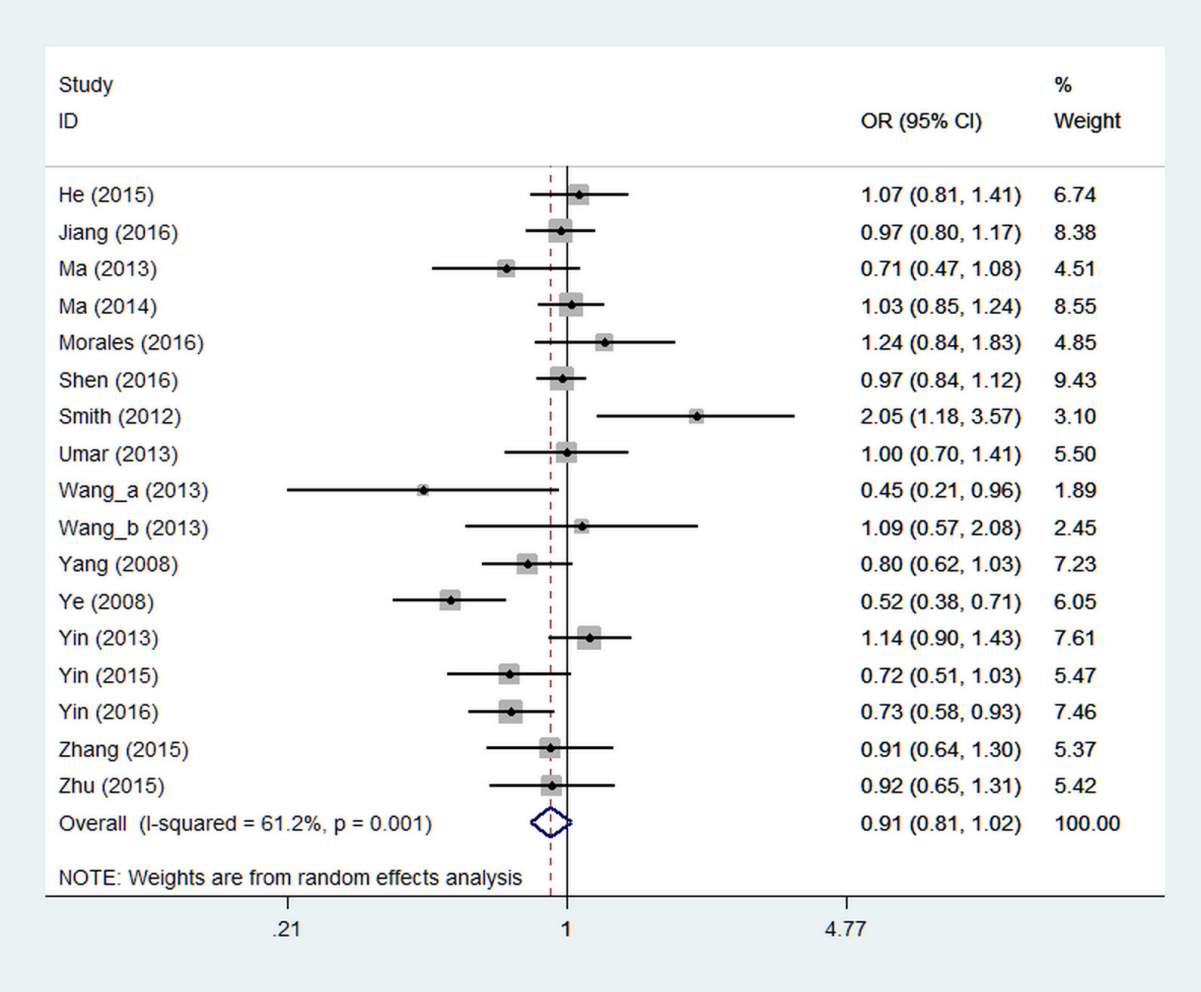
Forest plot of miR-423 rs6505162 and overall cancer risk under dominant model Notes: Wang_a (2013) means black population in South Africa, Wang_b (2013) means population of mixed ethnicities in South Africa.

**Figure 3:**
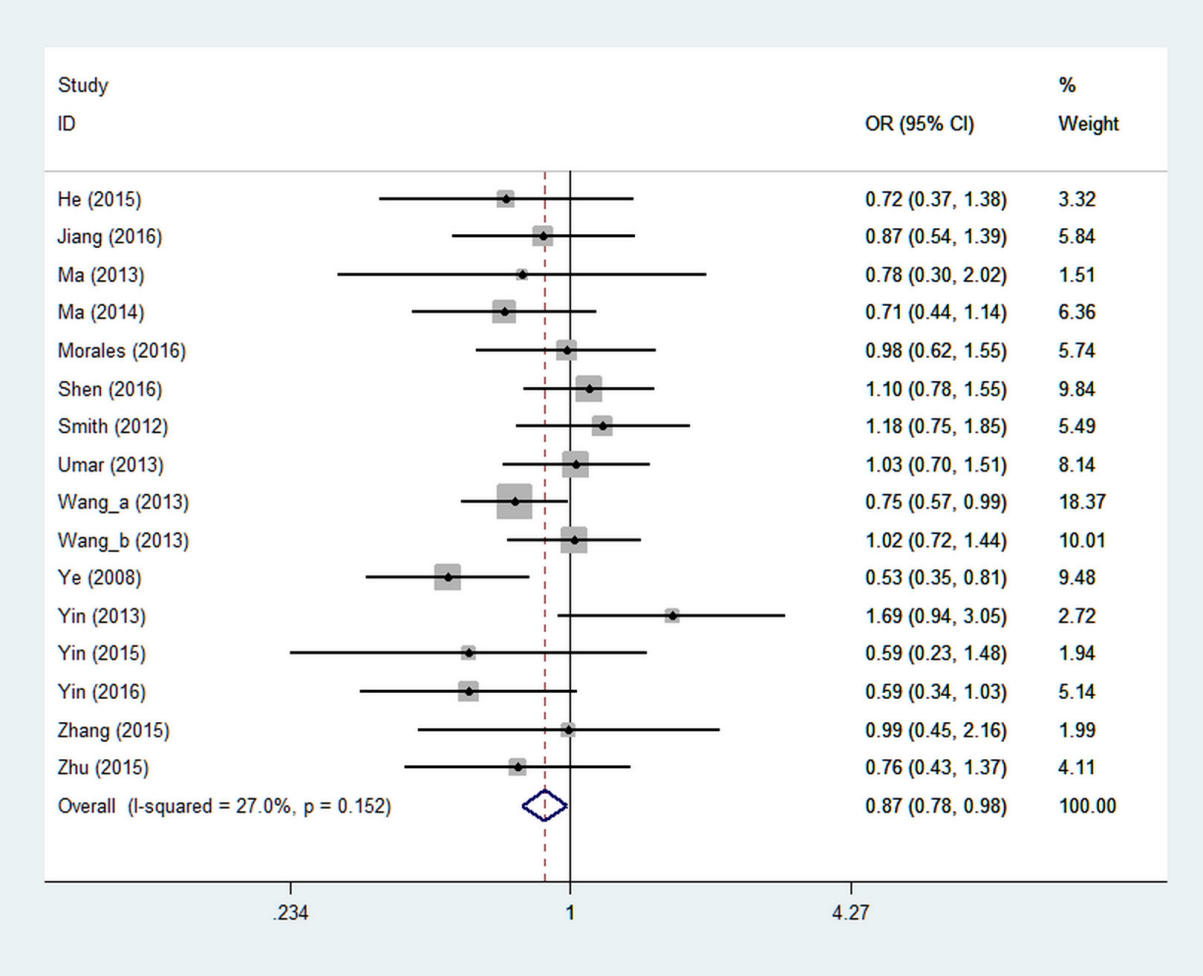
Forest plot of miR-423 rs6505162 and overall cancer risk under recessive model Notes: Wang_a (2013) means black population in South Africa, Wang_b (2013) means population of mixed ethnicities in South Africa.

Table 2 presents summary results concerning the association between miR-423 rs6505162 and the risk of overall cancer. Seventeen studies were included in the dominant model analysis and there were no significant associations observed with the odds ratio (OR) of 0.91 (95% confidence interval (CI): 0.81~1.02, *P* = 0.121, Figure 2). Due to the absence of AA-specific data in one study which indicated an increased risk of C allele, [32] sixteen studies were included in the recessive model analysis. The pooled results showed that AA genotype of miR-423 rs6505162 was associated with decreased cancer risk under recessive model (OR = 0.87, 95% CI: 0.78~0.98, *P* = 0.020) (Figure 3).

**Table 2:** Meta-analysis of the association between the miR-423 rs6505162 and overall cancer risk

Subgroup	Dominant model				Recessive model			
	No. of studies	Model	Odds ratio (95% CI)	*P* value	No. of studies	Model	Odds ratio (95% CI)	*P* value
Total	17	Random	0.91 (0.81–1.02)	0.121	16	Fixed	0.87 (0.78–0.98)	0.020
Ethnicity								
Asian	11	Fixed	0.95 (0.88–1.02)	0.161	11	Fixed	0.91 (0.78–1.06)	0.231
Caucasian	3	Random	0.91 (0.49–1.67)	0.754	2	Random	0.79 (0.36–1.73)	0.556
Black	1	–	0.45 (0.21–0.96)	0.039	1	–	0.75 (0.57–0.99)	0.039
Mixed	2	Fixed	1.20 (0.86–1.67)	0.293	2	Fixed	1.00 (0.76–1.32)	**0.981**
Cancer								
Breast cancer	5	Random	1.08 (0.82–1.42)	0.578	5	Fixed	0.98 (0.75–1.27)	0.859
Esophageal cancer	7	Random	0.87 (0.69–1.08)	0.206	7	Random	0.91 (0.72–1.15)	0.431
Lung cancer	2	Fixed	0.73 (0.60–0.89)	0.002	2	Fixed	0.59 (0.37–0.95)	0.031
Gastric cancer	1	–	0.97 (0.80–1.17)	0.718	1	–	0.87 (0.54–1.39)	0.559
Hepatocellular carcinoma	1	–	1.03 (0.85–1.24)	0.787	1	–	0.71 (0.44–1.14)	0.158
Bladder cancer	1	–	0.80 (0.62–1.73)	0.084	0*	–	–	–

Note: It was excluded in the recessive model analysis due to that the AA-specific data was not available in this study.

### Subgroup analysis

Subgroup analyses were performed based on ethnicity and cancer type (Table 2). There was a wide variation in the A allele frequency of miR-423 rs6505162 among cancer patients of different races. Eleven studies investigated the association in Asian populations (Chinese and Indian), with negative results observed under the dominant or the recessive model (OR = 0.95, 95% CI: 0.88~1.02, *P* = 0.161; OR = 0.91, 95% CI: 0.78~1.06, *P* = 0.231, respectively). With respect to cancer type, a significant decrease in cancer risk was found only in lung cancer patients (dominant model: OR = 0.73, 95% CI: 0.60~0.89, *P* = 0.002; recessive model: OR = 0.59, 95% CI: 0.37~0.95, *P* = 0.031). The results of meta-regression analysis showed that cancer types and ethnicity do not affect the association between miR-423 rs6505162 polymorphism and cancer susceptibility (both *P* > 0.05).

### Sensitivity analysis

Sensitivity analysis performed by excluding the “low quality” study with a NOS score of < 6 [22]. The pooled results showed no significant change for the recessive model (OR = 0.87, 95% CI: 0.77~0.97, *P* = 0.017), indicating that patients carrying miR-423 rs6505162 AA genotype may have a decreased cancer risk compared with those carrying the CC/AC genotype.

We also did another sensitivity analysis by excluding the study with the smallest OR in the recessive model [27]. The pooled result was significantly changed in the overall population (OR = 0.91, 95% CI: 0.81~1.02, *P* = 0.114).

In addition, 9 studies reported the adjusted ORs under dominant model, while 8 studies reported the adjusted ORs under recessive model (Table 1). The pooled ORs of dominant model analysis was 0.87 (95% CI: 0.75~0.99), and it was 0.87 (95% CI: 0.76–0.99) for recessive model analysis (Supplementary Figures 1 and 2).

### Publication bias

**Figure 4:**
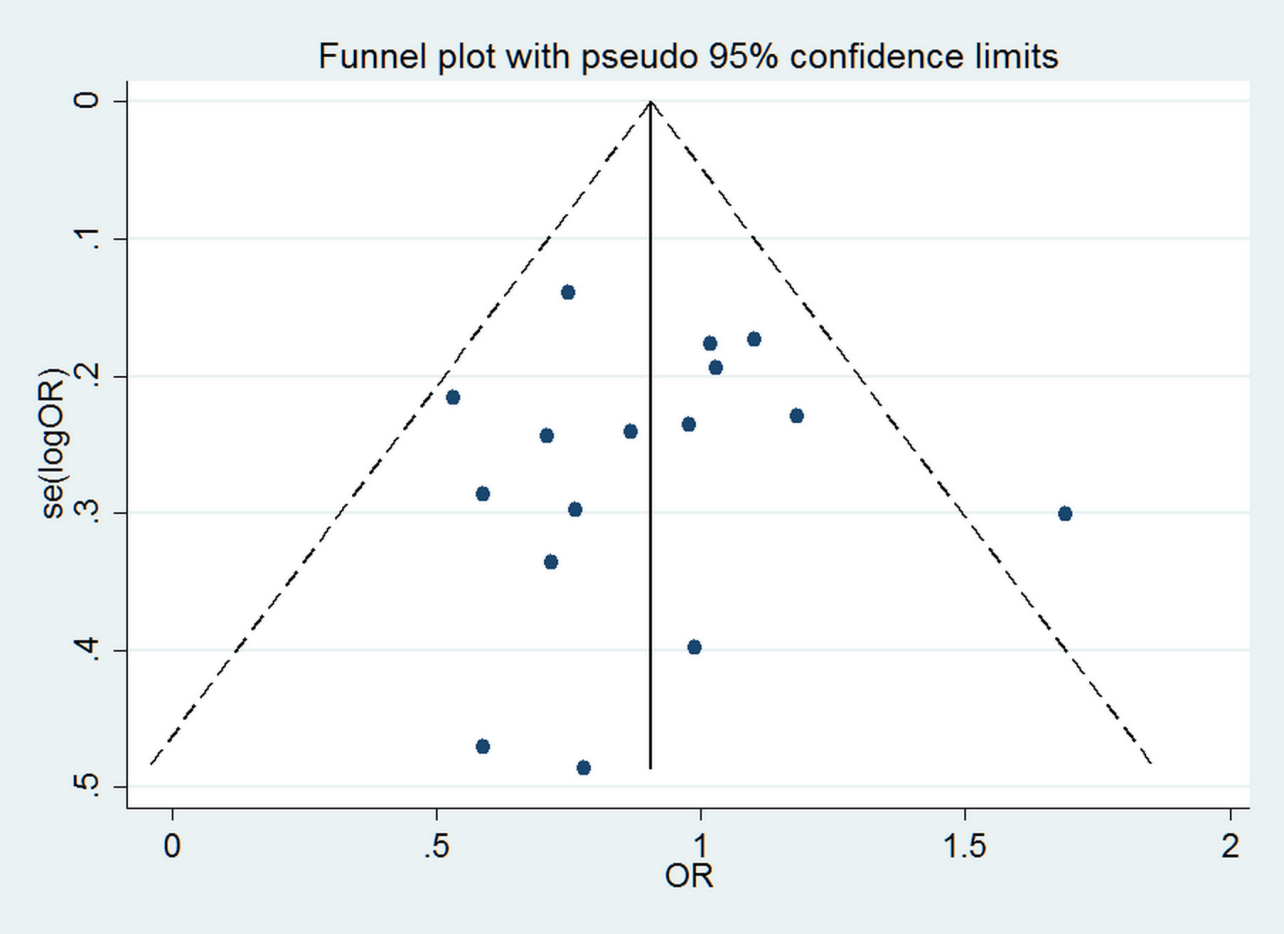
Funnel plot to detect publication bias under recessive genetic model

Visual inspection of funnel plots and Egger's test was used to evaluate the publication bias in our meta-analysis. Taking the recessive genetic model as an example, the funnel plot is displayed in Figure 4. The statistical results still showed there was no publication bias in our study (Egger's test *P* = 0.952 for the dominant model and *P* = 0.906 for the recessive model).

## DISCUSSION

To the best of our knowledge, this meta-analysis is the first to comprehensively evaluate the association between miR-423 rs6505162 (C > A) and all cancer risk. In this meta-analysis, 17 eligible case-control studies involving 8,582 cases and 10,291 controls were analyzed. Our major finding suggested that a significant association between miR-423 rs6505162 and cancer susceptibility was observed in recessive models. Subgroup analyses also linked the A allele with a significant decreased risk of lung cancer.

About seven of 17 included studies reported significant association between rs6505162 and risk of different cancers [22, 24, 26–28, 30]. Two studies concluded that the A allele of the rs6505162 increased the risk of breast cancer, [22, 24] whereas the same allele presented a decreased risk of developing lung cancer and bladder cancer in two other studies [30, 32]. The inconsistent effect for the A allele was also observed among three esophageal cancer studies [26–28]. Meta-analysis, as a powerful statistical method, could provide a quantitative approach for pooling the results of different studies on the same topic and estimating the diversity. After pooling all published studies into the meta-analysis, cancer risk associated with miR-423 rs6505162 allele was significant in the recessive model (AA vs AC/CC). Sensitivity analysis revealed that this association turned to be non-significant after excluding the study with the smallest OR. This is the first time analysis that conducted in a Caucasian population with 346 pairs of cases and controls. The result indicated that the A allele significantly reduced esophageal cancer risk with an OR of 0.64 (95% CI, 0.51~0.80) [27]. Then we conducted a power analysis based on a simple online tool (http://osse.bii.a-star.edu.sg/calculation2.php), assuming that the minor allele frequency (MAF) of the A allele is 0.2785 and 0.2980 in cases and controls, respectively, according to our own data. The power before and after excluding the above study were just 81% and 79% in corresponding sample size, respectively. Another sensitive analysis revealed that the pooled ORs of recessive model analysis kept significant, based on the 8 studies reported the adjusted ORs. With these evidences, as well as the almost marginal significance in original recessive model, the non-significant result (*P* = 0.114) in our sensitive analysis may be mostly due to the insufficient sample size. Furthermore, our result was consistent with another cross phenotype meta-analysis (CPMA), which is based on an *in silico* re-analysis of the human genotyping data downloaded from dbGAP [35].

The meta-analysis was also performed in different populations and cancer types to further eliminate heterogeneity. When stratified by race, no significant association was detected in any genetic model involving any population, but an exception was found for both the dominant model and the recessive model in a black population (both ORs < 1). However, interpreting this result should be done cautiously, due to the fact that the only included study had a limited sample size of about 368 cases and 583 controls [26]. During subgroup analysis of cancer types, the risk reduction of the A allele was only significant in the subgroup of lung cancer, based on two studies of Chinese populations [29, 30]. Hu et al. also presented an approaching significant protective effect of the A allele in about 3,800 pairs of Caucasians [35]. In addition, it is worth noting that we failed to get any significant result for either breast cancer or esophageal cancer. There were five breast cancer studies and seven esophageal cancer studies. However, both cancer types had relatively small effect sizes in our pooled data, which might explain the negative result. Our results were similar to two previous meta-analysis studies about breast cancer and esophageal cancer, which both just included a subset (two and four studies) of our study, respectively [36, 37]. In fact, the difference in magnitude and significance in our study in turn provided evidence for the heterogeneity among cancer types, and suggested a possible role of cancer differences. Given the non-significant result of meta-regression analysis, further attention should be paid to enlarge sample size and strengthen the statistical power for those cancers.

It is known that rs6505162 lies within the first intron of the gene of nuclear speckle splicing regulatory protein (*NSRP1*), [24, 38] and produces two mature transcripts designated miR-423-3p and miR-423-5p [13, 39]. However, the potential functions and exact mechanistic roles of miR-423 in cancer remain elusive, and inconsistent in different tumor types. For example, different expression patterns of miR-423 have been reported in various types of cancers, such as under-expression in mesothelioma, [14] oral cancer, [40] inconsistent result for colorectal cancer, [41–43] while there was over-expression in head and neck cancer, [15] laryngeal carcinoma, [44] female genital system neoplasms (breast, cervical and endometrial), [16, 45–47] and most of the digestive system neoplasms (gastric, pancreatic, hepatocellular) [48–50, 51]. Some studies indicated that miR-423 acts as tumor suppressor in oral cancer, [40] as oncogene in hepatocellular carcinoma, [44, 51] but inconsistent results in breast cancer [52, 53]. Additionally, only two studies suggested that the C to A substitution in rs6505162 promotes the production of mature *miR-423* in cell lines from breast cancer and endometrial carcinoma, [53, 54] *but the SNP* was not correlated with expression of miR-423 in esophageal squamous cell carcinoma [26]. Moreover, it is still unclear whether modulations of mature levels of miR-423 are functionally linked to this SNP in many other cancers [8]. Third, overexpression of miR-423 was observed to promote cell proliferation in colorectal cancer, [41] and gastric cancer, [48] but it inhibited cell proliferation in endometrial carcinoma [54]. The proposed target genes also seem quite different for different cancers, e.g. *TIF1* (gastric cancer), [48] *ATG7* (hepatocarcinoma), [55] and *KLF2* (ovarian cancer) [47]. All these conflicting data and uncertain evidence remind us again of the significant heterogeneity among cancer types [8]. Tumor-specific functional studies are especially needed to clarify biological effects of the tissue heterogeneity on the expression and function of miR-423 and to experimentally validate its potential targets, so as to illustrate the underlying mechanism and interpret the data appropriately.

We believe that this is the first quantitative assessment focused on the association between miR-423 rs6505162 alleles and all types of cancer. Our results are reliable for the following reasons. First, the genotype distributions in the controls of this SNP were all mostly consistent with HWE. Second, no apparent publication bias was observed by either Begg's funnel plot or Egger's test. Third, all included studies used high quality genotyping methods according to the methodological quality assessment. Some limitations of this study should be also acknowledged. As noted, only 16 articles with 17 studies were included, and the data in some analyses and subgroup analyses (e.g. ethnicity, cancer types) were relatively insufficient. Almost 70% of the participants included in this meta-analysis were from one ethnical group (Chinese); therefore generalizations should be made cautiously. The lack of original data limited further analysis of the potential interactions. Finally, although the statistical tests for publication bias were not significant, publication bias may still exist due to that studies with negative results often have less chance for publication. Considering all these factors, our results should be interpreted with caution, but we believe these findings could help to explain the association between miR-423 rs6505162 and cancer risk.

In conclusion, this meta-analysis indicated that miR-423 rs6505162 C > A may reduce the risk of cancer, especially for lung cancer. The high heterogeneity among cancer types indicated that this polymorphism might play different roles in different cancers. However, this finding needs to be evaluated further in larger samples, especially for subgroup analyses. In addition, cancer-specific functional characterizations are simultaneously needed to reveal the underlying mechanisms between miR-423 rs6505162 and the etiology of cancer.

## MATERIALS AND METHODS

### Literature research

A comprehensive search was conducted to identify all eligible publications in PubMed and Embase electronic databases as of 10th September, 2016. The medical subject headings (MeSH) and free-text words were used. Search terms mainly included (“MIRNA423 microRNA, human” [Supplementary Concept] OR mir-423 OR microrna-423 OR mir423 OR rs6505162) AND (“Carcinoma”[Mesh] OR “Neoplasms”[Mesh] OR malignancy OR tumor OR neoplasia OR carcinoma OR Cancer). We also carefully checked references of the retrieved articles to find additional eligible studies. During the course of literature search, no language or other limits were set.

### Inclusion and exclusion criteria

The study was considered eligible if it met the following criteria: (1) designed as case-control or cohort study, (2) evaluated the association between miRNA-423 polymorphism and cancer risk, (3) provided sufficient data (the numbers of genotypes distribution in two groups, respectively) for calculating the OR and its 95% CI. We excluded letters, comments, correspondence, conference reports and laboratory studies or articles that did not contain enough data with which to compute the ORs.

### Data extraction

Two investigators independently extracted the following items from each eligible study: surname of first author, country of the investigation, year of publication, ethnicity, genotyping method, genotype distribution and HWE in the control group. A discussion was carried out to achieve consensus when discrepancies were noted.

### Methodological quality assessment

Two investigators independently evaluated the quality of eligible studies using the NOS, [56] which was one of the most commonly used tools for assessing quality of observational studies in a meta-analysis setting. The NOS encompasses three parts, i.e. case and control selection, comparability, and exposure. Each of them respectively comprises four, two, and three items. Each item is given one point, with nine points in total. If the study got fewer than six points, it would be regarded as “low quality”; otherwise, it would be regarded as “high quality”. Disagreements between reviewers regarding data extraction were resolved through discussion.

### Data analysis

Crude ORs together with their corresponding 95% CIs were calculated to assess the strength of association between miRNA-423 rs6505162 and overall cancer risk under dominant and recessive models. HWE was examined for each study by the Chi-square test in the control groups, and *P* < 0.05 was considered a significant departure from the HWE. The I^2^ statistic and *Q* test were used to measure the between-study heterogeneity. If I^2^ < 50% and *P* > 0.1, the heterogeneity was considered mild, and the summary ORs were combined under a fixed-effects model, otherwise a random-effects model were used. The *Z* test was used to assess the statistical significance of pooled ORs, and two-tailed *P*-values < 0.05 were considered significant.

Both subgroup analyses and meta-regression analysis were performed to explore potential sources of heterogeneity in ethnicity and cancer types. Sensitivity analyses were performed in two steps: (1) excluding the studies with “low quality”, and (2) excluding the studies with the biggest or smallest OR in genetic models with statistically significant findings. Furthermore, we also extracted the adjusted OR and 95% CIs from the original literature to evaluate the stability of the findings. Visual inspection of funnel plots and Egger's regression asymmetry test were applied to assess potential publication bias. STATA 14.0 (Stata Corporation, College Station, Texas, USA) was used for statistical analyses.

## SUPPLEMENTARY MATERIALS FIGURES


