# Supplementary material for: The association between miR-423 rs6505162 polymorphism and cancer susceptibility: a systematic review and meta-analysis
Source: Oncotarget. 2017 Mar 17;8(25):40204–13. doi: 10.18632/oncotarget.16319 (PMC5522323; doi:10.18632/oncotarget.16319)
Supplement: Supplementary file 1 [file oncotarget-08-40204-s001.pdf]

The association between miR-423 rs6505162 polymorphism and cancer susceptibility: a systematic review and meta-analysis

Supplementary Materials

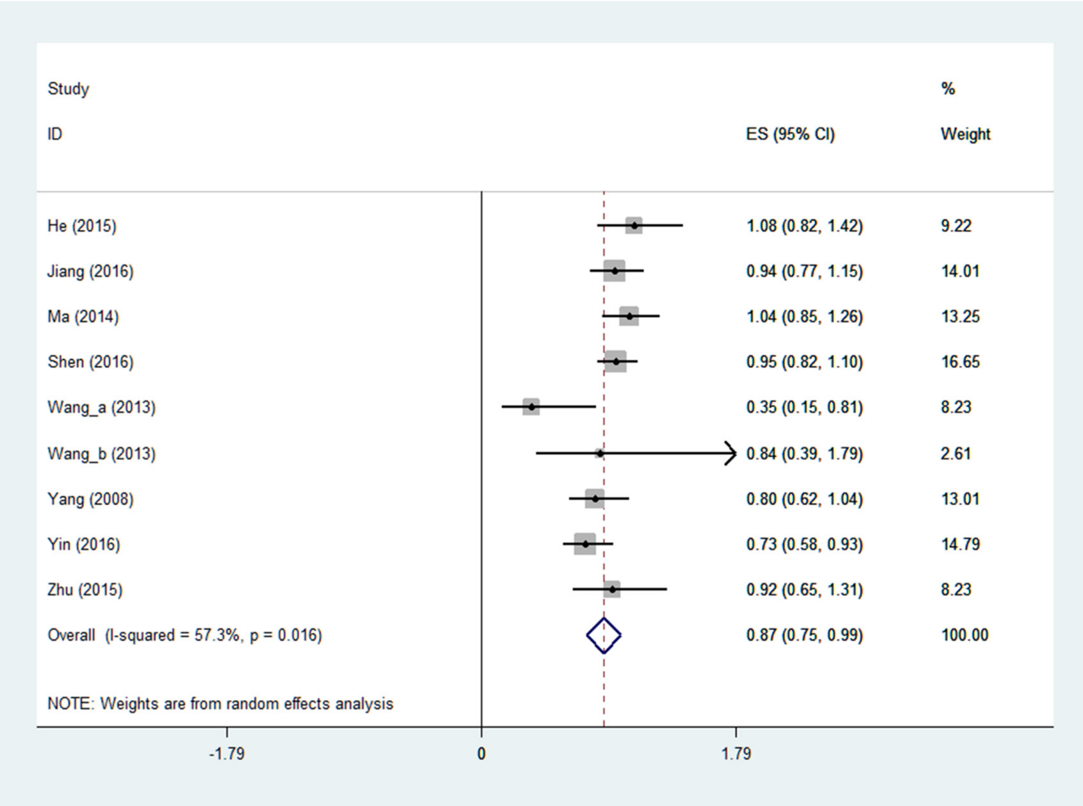

Supplementary Figure 1: Forest plot of miR-423 rs6505162 and overall cancer risk under dominant model (based on reported adjusted odds ratios). Notes: Wang_a (2013) means black population in South Africa, Wang_b (2013) means population of mixed ethnicities in South Africa.

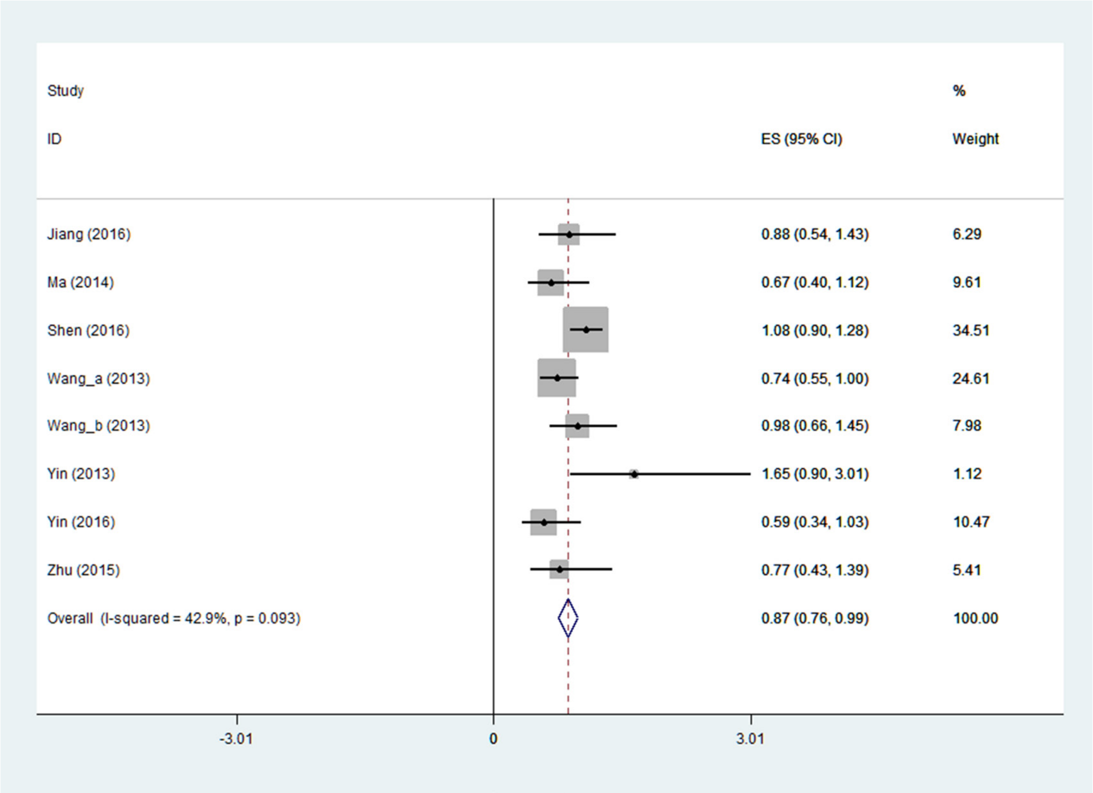

Supplementary Figure 2: Forest plot of miR-423 rs6505162 and overall cancer risk under recessive model (based on reported adjusted odds ratios). Notes: Wang_a (2013) means black population in South Africa, Wang_b (2013) means population of mixed ethnicities in South Africa.
